# Alcohol Consumption and Colorectal Cancer Risk: Findings from the JACC Study

**DOI:** 10.2188/jea.15.S173

**Published:** 2005-08-18

**Authors:** Kenji Wakai, Masayo Kojima, Koji Tamakoshi, Yoshiyuki Watanabe, Norihiko Hayakawa, Koji Suzuki, Shuji Hashimoto, Miyuki Kawado, Shinkan Tokudome, Sadao Suzuki, Kotaro Ozasa, Hideaki Toyoshima, Yoshinori Ito, Akiko Tamakoshi

**Affiliations:** 1 Division of Epidemiology and Prevention, Aichi Cancer Center Research Institute.; 2 Department of Health Promotion and Preventive Medicine, Nagoya City University Graduate School of Medical Sciences.; 3 Department of Public Health/Health Information Dynamics, Nagoya University Graduate School of Medicine.; 4 Department of Epidemiology for Community Health and Medicine, Kyoto Prefectural University of Medicine Graduate School of Medical Science.; 5 Department of Epidemiology, Research Institute for Radiation Biology and Medicine, Hiroshima University.; 6 Department of Public Health, Fujita Health University School of Health Sciences.; 7 Department of Hygiene, Fujita Health University School of Medicine.; 8 Department of Preventive Medicine/Biostatistics and Medical Decision Making, Nagoya University Graduate School of Medicine.

**Keywords:** Alcohol Drinking, Colon Neoplasms, Rectal Neoplasms, Cohort studies

## Abstract

BACKGROUND: Because alcohol drinking is a potential risk factor for colorectal cancer, the trend in alcohol consumption in Japan may partly explain the increase in incidence and mortality rates of this malignancy until 1990-1995.

METHODS: We analyzed data from the Japan Collaborative Cohort Study. From 1988 to 1990, 23,708 men and 34,028 women, aged 40-79 years, completed a questionnaire on lifestyle factors including drinking habits. Incidence rate ratios (IRR) were estimated by using proportional hazards models.

RESULTS: During the mean follow-up of 7.6 years through December 1997, we documented 418 incidents of colon cancer and 211 of rectal cancer. Male ex- or current drinkers demonstrated a twofold risk for colon cancer compared with nondrinkers: the multivariate-adjusted IRR was 2.01 (95% confidence interval [CI] 1.09-3.68) for ex-drinkers and 1.97 (95% CI: 1.28-3.03) for current drinkers. The dose-response relationship between alcohol consumption and the risk, however, was not clear. Female ex-drinkers were at an increased risk without statistical significance. For rectal cancer, we found a slightly lower risk in light current drinkers who consumed less than 22 g ethanol per day: the multivariate IRR was 0.61 (95% CI: 0.33-1.13) for men and 0.69 (95% CI: 0.27-1.74) for women. Although the IRR for all current drinkers was almost unity in men, an increasing trend in risk was detected with increasing alcohol consumption in current drinkers (trend p = 0.027).

CONCLUSIONS: Taking the findings from our study and other prospective investigations into consideration, more attention should be paid to alcohol consumption in the prevention of colon cancer in Japan.

The age-adjusted incidence and mortality rates of colorectal cancer in Japan increased linearly until 1990-1995, and thereafter leveled off.^1^^,^^2^ These trends have been generally ascribed to changes in diet, particularly to an increase in fat or meat consumption and a decrease in the intake of dietary fiber.^3^^-^^5^

Alcohol consumption, however, also grew rapidly in Japan before the 1990s. Because drinking is a potential risk factor for colorectal cancer,^6^^,^^7^ this increase in consumption may partly explain the increase in incidence and mortality rates of this malignancy until 1990-1995. Nevertheless, little attention has been paid to drinking habits in relation to the prevention of colorectal cancer in Japan compared with dietary factors.

We therefore examined the association of drinking with the risk of colorectal cancer, using the dataset from the Japan Collaborative Cohort Study (JACC Study) for Evaluation of Cancer Risk sponsored by the Ministry of Education, Science, Sports and Culture of Japan (Monbusho), a nation-wide prospective study.

## METHODS

### Study Cohort

The JACC Study started in 1988 to 1990, when 110,792 inhabitants aged 40 to 79 years completed a baseline questionnaire.^8^^,^^9^ They were enrolled from 45 study areas throughout Japan, mostly as they underwent municipal health check-ups. Informed consent for participation was obtained individually from each participant, except in a few study areas where informed consent was provided at the group level after the aim of the study and confidentiality of the data had been explained to community leaders. The Ethics Committee of Medical Care and Research of Fujita Health University approved this investigation.

Subjects for the present analysis were restricted to 61,557 individuals who lived in 22 study areas, where information on cancer incidence is available, and questions to estimate alcohol intake were included in the questionnaire. Of the total, we excluded 57 with a previous history of colorectal cancer and 3,764 of unknown drinking status (nondrinkers, ex-drinkers, or current drinkers), leaving 57,736 subjects (23,708 men and 34,028 women) for the analysis.

### Drinking Habits and Other Exposure Data

The baseline questionnaire covered lifestyle factors including smoking and drinking habits, physical activity, and consumption of selected foods, as well as medical history, education, family history of cancer, height and weight, and occupation held the longest. For alcohol intake, subjects were asked to report their drinking status (nondrinkers, ex-drinkers, or current drinkers). Ex-drinkers or current drinkers were asked the frequency of alcohol consumption with four possible responses: less than once/week, 1-2 times/week, 3-4 times/week, or almost every day. They also report the average intake at each time in a Japanese drink (‘gou’). One Japanese drink is equivalent to 22 g ethanol. We estimated daily alcohol intake by multiplying the average amount on each occasion by the frequency of drinking alcoholic beverages.

### Follow-up

We used population registries in the municipalities to determine the vital and residential status of subjects. We ascertained the incidence of cancer by means of linkage with the records of population-based cancer registries, supplemented by a systematic review of death certificates.^8^ In some study areas, medical records in local major hospitals were also reviewed. The follow-up was conducted from the time of the baseline survey through the end of 1997 except in one area (to the end of 1994). During the study period, only 3.3% (n = 1,921) of the participants were lost to follow-up due to relocation.

The mortality-to-incidence ratio for colorectal cancer was 0.28 in the cohort covered by cancer registries. This figure is comparable with those in acceptably-accurate population-based cancer registries in Japan (0.23 to 0.51),^10^ and indicates that a reasonably high proportion of colorectal cancer cases were identified.

### Statistical Analysis

We categorized subjects into groups by drinking status and alcohol consumption and compared background characteristics between the groups by the one-way analysis of variance or the *χ*^2^ test.

We counted person-time of follow-up for each participant from the date of filling out the baseline questionnaire to the development of colorectal cancer, death from any cause, emigration to outside the study area, or the end of the follow-up period, whichever came first. Those who died from causes other than colorectal cancer or moved out of the study areas were treated as censored cases.

The incidence rate ratios (IRR) for colon or rectal cancer according to drinking status and alcohol intake at baseline were estimated by gender using proportional hazards models,^11^ with adjustment for age and other potential confounders.^6^ The level of alcohol consumption in current drinkers was categorized into four groups in men (0.0-0.9, 1.0-1.9, 2.0-2.9, and 3.0+ Japanese drinks/day [=0-153,154-307, 308-461, and 462+ g ethanol/week]) and two groups in women (0.0-0.9 and 1.0+ Japanese drinks/day [= 0-153 and 154+ g ethanol/week]). The potential confounding factors adjusted included area (Hokkaido and Tohoku, Kanto, Chubu, Kinki, Chugoku, or Kyushu), education (attended school until the age of <16, 16-18, or 19+), family history of colorectal cancer in parents or siblings (yes or no), body mass index (<20.0, 20.0-24.9, or 25.0+ kg/m^2^ for men, and <20.0, 20.0-24.9, 25.0-29.9, or 30.0+ kg/m^2^ for women), smoking habits (never smokers, ex-smokers, or current smokers), walking time (≤30 or 30+ minutes/day), sedentary work (yes or no), and consumption of green leafy vegetables (≤2 times/week, 3-4 times/week, or every day), and beef (almost never, 1-2 times/month, 1-2 times/week, or 3+ times/week). Missing values for each covariate were treated as an additional category of the variable and were included in the proportional hazards model.

The dose-response relationship among current drinkers was assessed by the regression model assigning scores (0, 1, 2, or 3) to the levels of alcohol consumption. All p values were two-sided, and all the analyses were performed using the Statistical Analysis System^®^.^12^

## RESULTS

During the mean follow-up of 7.6 (standard deviation 1.9) years, we identified 418 incident cases of colon cancer (220 in men and 198 in women) and 211 cases of rectal cancer (150 in men and 61 in women).

Table 1 summarizes the background characteristics of the subjects according to drinking habits by gender. At baseline, nondrinkers, ex-drinkers, and current drinkers accounted for 18.5%, 6.9%, and 74.5% of men and 74.3%, 1.9%, and 23.8% of women, respectively (Daily alcohol consumption was unknown in some of the current drinkers). Heavy drinkers (3.0+ Japanese drinks/day) in men and moderate to heavy drinkers (1.0+ Japanese drinks/day) in women tended to be younger and less educated, are likely to be current smokers, and consume less green leafy vegetables and more beef. Ex-drinkers in both genders tended to be older and not to have been engaged in sedentary work. The proportion of current smokers was relatively high in female former drinkers.

**Table 1.**
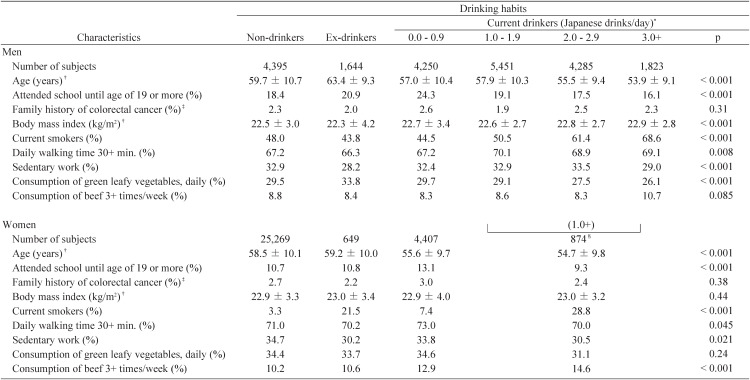
Background characteristics of subjects according to drinking habits by sex.

* : One Japanese drink (‘gou’) is equivalent to 22 g ethanol. The categories of 0.0-0.9, 1.0-1.9, 2.0-2.9, 3.0+, and 1.0+ Japanese drinks/day correspond to those of 0-153, 154-307, 308-461, 462+, and 154+ g ethanol/week.

† : Values are means ± standard deviation.

‡ : Family history in parents and/or siblings.

§ : Women who consumed 1.0+ Japanese drinks/day are grouped into one category.

Male ex- or current drinkers demonstrated a twofold risk for colon cancer compared with nondrinkers: the multivariate-adjusted IRR (IRR2 in Table 2) was 2.01 (95% CI: 1.09-3.68) for ex-drinkers and 1.97 (95% CI: 1.28-3.03) for current drinkers. The dose-response relationship between alcohol consumption and the risk, however, was not clear. Female ex-drinkers were at a somewhat increased risk, but it was far from significant.

**Table 2.** Incidence rate ratios (IRR) for cancers of the colon and rectum according to drinking habits at baseline by sex.

Sex	Drinking habits	Person-years	Colon					Rectum				
	
			No. of cases	IRR1*	95% CI^†^	IRR2*	95% CI^†^	No. of cases	IRR1*	95% CI^†^	IRR2^‡^	95% CI^†^
Men	Nondrinkers	33,018	24	1.00	(reference)	1.00	(reference)	30	1.00	(reference)	1.00	(reference)
	Ex-drinkers	11,291	19	2.01	1.10 - 3.67	2.01	1.09 - 3.68	14	1.19	0.63 - 2.24	1.25	0.66 - 2.38
	Current drinkers^§^	135,710	177	2.10	1.37 - 3.23	1.97	1.28 - 3.03	106	1.01	0.67 - 1.51	1.01	0.67 - 1.52
	0.0-0.9 (Japanese drinks/day)^||^	32,636	43	2.09	1.27 - 3.45	2.01	1.22 - 3.33	16	0.62	0.34 - 1.14	0.61	0.33 - 1.13
	1.0-1.9	41,446	63	2.32	1.45 - 3.71	2.22	1.38 - 3.56	35	1.02	0.63 - 1.67	1.01	0.62 - 1.65
	2.0-2.9	33,315	36	1.87	1.11 - 3.15	1.75	1.04 - 2.96	29	1.18	0.71 - 1.98	1.21	0.72 - 2.04
	3.0+	14,145	20	2.68	1.47 - 4.88	2.40	1.31 - 4.40	12	1.25	0.63 - 2.47	1.32	0.67 - 2.63
				Trend p = 0.76^¶^		Trend p = 0.85^¶^			Trend p = 0.038^¶^		Trend p = 0.027^¶^	

Women	Nondrinkers	193,562	149	1.00	(reference)	1.00	(reference)	50	1.00	(reference)	1.00	(reference)
	Ex-drinkers	4,573	6	1.64	0.73 - 3.71	1.56	0.68 - 3.60	1	0.82	0.11 - 5.92	0.78	0.11 - 5.78
	Current drinkers^§^	58,957	43	1.13	0.81 - 1.60	1.03	0.72 - 1.45	10	0.74	0.37 - 1.46	0.71	0.35 - 1.42
	0.0-0.9 (Japanese drinks/day)^||^	32,068	22	1.12	0.72 - 1.76	1.06	0.67 - 1.68	5	0.69	0.28 - 1.75	0.69	0.27 - 1.74
	1.0+	6,253	5	1.39	0.57 - 3.38	1.22	0.49 - 3.03	2	1.48	0.36 - 6.11	1.53	0.36 - 6.47
				Trend p = 0.64^¶^		Trend p = 0.96^¶^			Trend p = 0.36^¶^		Trend p = 0.36^¶^	

* : IRR1: adjusted for age.

† : CI: confidence interval.

‡ : IRR2: adjusted for age, area (Hokkaido and Tohoku, Kanto, Chubu, Kinki, Chugoku, or Kyushu), education (attended school until the age of < 16, 16-18, or 19+), family history of colorectal cancer in parents or siblings (yes or no), body mass index (< 20.0, 20.0-24.9, or 25.0+ kg/m^2^ for men, and < 20.0, 20.0-24.9, 25.0-29.9, or 30.0+ kg/m^2^ for women), smoking habits (never smokers, ex-smokers, or current smokers), walking time (≤ 30 or 30+ minutes/day), sedentary work (yes or no), and consumption of green leafy vegetables (≤ 2 times/week, 3-4 times/week, or every day), and beef (almost never, 1-2 times/month, 1-2 times/week,or 3+ times/week).

§ : Including subjects with unknown alcohol consumption.

|| : One Japanese drink (‘gou’) is equivalent to 22 g ethanol. The categories of 0.0-0.9, 1.0-1.9, 2.0-2.9, 3.0+, and 1.0+ Japanese drinks/day correspond to those of 0-153, 154-307, 308-461, 462+, and 154+ g ethanol/week.

¶ : The test for a trend in current drinkers.

For rectal cancer, we found a slightly lower risk in light current drinkers who consumed less than one Japanese drink/day in both genders: the IRR2 was 0.61 (95% CI: 0.33-1.13) for men and 0.69 (95% CI: 0.27-1.74) for women. Although the IRR2 for all current drinkers was almost unity in men, an increasing trend in risk was detected with an increasing alcohol consumption in current drinkers (trend p = 0.027). The IRR2 was 0.61, 1.01, 1.21, and 1.32 for <1.0, 1.0-1.9, 2.0-2.9, and 3.0+ Japanese drinks/day, respectively.

The age-adjusted IRRs (IRR1) were almost the same as the multivariate rate ratios (IRR2). We repeated the analyses in Table 2 after excluding the first two years of follow-up from the risk period but the findings remained materially unchanged. The IRR2 for colon cancer was 2.07 (95% CI: 1.05-4.10) in male ex-drinkers, 1.94 (95% CI: 1.19-3.15) in male current drinkers, 1.64 (95% CI: 0.66-4.11) in female ex-drinkers, and 1.01 (95% CI: 0.68-1.50) in female current drinkers. The corresponding figures for rectal cancer were 1.46 (95% CI: 0.72-2.95) for male ex-drinkers, 0.95 (95% CI: 0.60-1.51) for male current drinkers, and 0.89 (95% CI: 0.42-1.87) for female current drinkers. No case of rectal cancer was found in female former drinkers in this analysis.

## DISCUSSION

In a large-scale prospective study, we observed an association of drinking habits with the risk of colon cancer in men. A dose-response relationship in current drinkers, however, was not evident. For rectal cancer, a “J-shaped” association was found between alcohol intake and the risk, that is, the rate ratio was lowest in light drinkers.

In Japan, per adult ethanol consumption increased from 5.86 L/year in 1965 to 8.30 L/year in 1999 (available at http://www.ncc.go.jp/jp/statistics/2003/index.html). The alcohol drinking has been common in men and has also become more popular in women.^13^ If alcohol intake actually enhances the risk of colorectal cancer, therefore, its attributable risk will be large, and controlling alcohol drinking will be of great importance for the primary prevention of this cancer in Japan. Given that male former or current drinkers have twice the risk of colon cancer as nondrinkers, and the distribution of drinking habits in our cohort is applicable to the general Japanese population, the population attributable risk percent would be 45%. This may imply that nearly half of all male colon cancer is ascribable to alcohol drinking.

Alcohol intake can cause malabsorption of folate and block its release from the hepatocyte. In addition, alcohol metabolite acetaldehyde may inactivate methyltetrahydrofolate or inhibit methionine synthase. These anti-folate effects may lead to DNA hypomethylation.^7^ Alcohol may also have specific carcinogenic effects; the colonic bacteria can produce substantial levels of acetaldehyde at ethanol concentrations that are common in the colonic mucosa of drinkers.^7^

A positive association of drinking habits with the risk of colon or colorectal cancer has been rather consistently found in cohort studies in Japan, particularly in men. Hirayama^14^ reported an increasing risk of cancer of the sigmoid colon with the increasing frequency of alcohol consumption in his Six-Prefecture Cohort Study: the relative risks in men compared with nondrinkers were 2.03, 3.83, and 5.42 for infrequent, occasional, and daily drinkers, respectively. Murata et al.^15^ also report a significantly elevated risk of colon cancer among alcohol drinkers in a male cohort, although without a clear dose-response relation.

In the Japan Public Health Center-based prospective study,^16^ another population-based cohort study, regular drinking of 150+ g/week of ethanol (about 1+ Japanese drinks/day) showed an increased risk of colorectal cancer only in men: relative risks compared with nondrinkers were 1.4 for 150-299 g/week and 2.1 for 300+ g/week. In a cohort of the Takayama Study,^17^ a positive dose-response relationship between alcohol consumption and colon cancer risk was observed for men and women. Thus, our study may well add further evidence for the role of alcohol intake in the development of colon cancer. Female current drinkers may not be associated with colorectal cancer because of the less intake of alcohol in our population; the median intake in the category of 0.0-0.9 Japanese drinks/day was 0.32 drinks in men and 0.15 drinks in women.

However, the decreased risk of rectal cancer in light drinkers in the present study does not seem to be supported by previous reports. One possibility is that the light drinkers had unknown characteristics that confounded the association between alcohol consumption and the risk. We may have failed to exclude all the confounding factors, although we adjusted for selected risk or protective factors, and the adjustment did not substantially alter the results.

The higher risk for colon cancer than for rectal cancer was observed in three prospective studies in Japan^14^^,^^15^^,^^17^ but not in the Japan Public Health Center-based prospective study.^16^ A recent pooled analysis of eight cohort studies in Western countries did not show a substantial difference between cancer of the colon and rectum.^18^ Further investigations would be warranted to elucidate if the contribution of alcohol differs between colon and rectal cancers in Japan.

The strengths of the present study are its prospective design and large size. We assessed drinking habits before the diagnosis of colorectal cancer; thus any errors of recall should have been non-differential between cases and non-cases. A considerable number of cases of colon and rectal cancers were identified in male current drinkers, which made it possible to assess the risk by the level of alcohol consumption.

Some methodological limitations, however, need consideration. First, the frequency and amount of alcohol consumption were based on self-reporting and may be subject to misclassification. In addition, we could not compute daily intake for some subjects due to their incomplete responses to the questionnaire. These issues in estimating alcohol intake may partly explain why we failed to uncover the dose-response relationship.

Second, information on drinking habits was collected only at baseline. If drinkers at baseline stopped drinking during the follow-up, it would have resulted in a somewhat attenuated risk for current drinkers. Although most cohort studies on drinking and the risk of colorectal cancer did not update data on the drinking habits of subjects, repeated measurements may provide more meaningful information. Finally, the number of rectal cancer cases among male and female ex-drinkers and female current drinkers was rather small as was the number of colon cancer cases in female ex-drinkers. We therefore cannot exclude an increase or a decrease in risk associated with alcohol consumption in these groups.

In conclusion, taking the findings from our study and other prospective investigations in Japan and the high percentage of male drinkers into consideration, more attention should be paid to alcohol consumption in the primary prevention of colon cancer in this country.

## MEMBER LIST OF THE JACC STUDY GROUP

The present investigators involved, with the co-authorship of this paper, in the JACC Study and their affiliations are as follows: Dr. Akiko Tamakoshi (present chairman of the study group), Nagoya University Graduate School of Medicine; Dr. Mitsuru Mori, Sapporo Medical University School of Medicine; Dr. Yutaka Motohashi, Akita University School of Medicine; Dr. Ichiro Tsuji, Tohoku University Graduate School of Medicine; Dr. Yosikazu Nakamura, Jichi Medical School; Dr. Hiroyasu Iso, Institute of Community Medicine, University of Tsukuba; Dr. Haruo Mikami, Chiba Cancer Center; Dr. Yutaka Inaba, Juntendo University School of Medicine; Dr. Yoshiharu Hoshiyama, University of Human Arts and Sciences; Dr. Hiroshi Suzuki, Niigata University School of Medicine; Dr. Hiroyuki Shimizu, Gifu University School of Medicine; Dr. Hideaki Toyoshima, Nagoya University Graduate School of Medicine; Dr. Kenji Wakai, Aichi Cancer Center Research Institute; Dr. Shinkan Tokudome, Nagoya City University Graduate School of Medical Sciences; Dr. Yoshinori Ito, Fujita Health University School of Health Sciences; Dr. Shuji Hashimoto, Fujita Health University School of Medicine; Dr. Shogo Kikuchi, Aichi Medical University School of Medicine; Dr. Akio Koizumi, Graduate School of Medicine and Faculty of Medicine, Kyoto University; Dr. Takashi Kawamura, Kyoto University Center for Student Health; Dr. Yoshiyuki Watanabe, Kyoto Prefectural University of Medicine Graduate School of Medical Science; Dr. Tsuneharu Miki, Graduate School of Medical Science, Kyoto Prefectural University of Medicine; Dr. Chigusa Date, Faculty of Human Environmental Sciences, Mukogawa Women’s University ; Dr. Kiyomi Sakata, Wakayama Medical University; Dr. Takayuki Nose, Tottori University Faculty of Medicine; Dr. Norihiko Hayakawa, Research Institute for Radiation Biology and Medicine, Hiroshima University; Dr. Takesumi Yoshimura, Fukuoka Institute of Health and Environmental Sciences; Dr. Akira Shibata, Kurume University School of Medicine; Dr. Naoyuki Okamoto, Kanagawa Cancer Center; Dr. Hideo Shio, Moriyama Municipal Hospital; Dr. Yoshiyuki Ohno, Asahi Rosai Hospital; Dr. Tomoyuki Kitagawa, Cancer Institute of the Japanese Foundation for Cancer Research; Dr. Toshio Kuroki, Gifu University; and Dr. Kazuo Tajima, Aichi Cancer Center Research Institute.
